# Growth-Promoting Effects of Grass Root-Derived Fungi *Cadophora fastigiata*, *Paraphoma fimeti* and *Plectosphaerella cucumerina* on Spring Barley (*Hordeum vulgare*) and Italian Ryegrass (*Lolium multiflorum*)

**DOI:** 10.3390/microorganisms13010025

**Published:** 2024-12-26

**Authors:** Izolda Pašakinskienė, Violeta Stakelienė, Saulė Matijošiūtė, Justas Martūnas, Marius Rimkevičius, Jurga Būdienė, Algis Aučina, Audrius Skridaila

**Affiliations:** 1 Botanical Garden, Vilnius University, Kairėnų 43, 10239 Vilnius, Lithuania; 2 Life Sciences Center, Vilnius University, Saulėtekio 7, 10221 Vilnius, Lithuania; 3 Nature Research Centre, Institute of Ecology, Akademijos Str. 2, 08412 Vilnius, Lithuania

**Keywords:** seed inoculation, growth stimulation, endophytic fungi, fungal volatile organic compounds (VOCs), plate-in-plate arrays, cereals, grasses

## Abstract

Many endophytic fungi are approved as plant growth stimulants, and several commercial biostimulants have already been introduced in agricultural practice. However, there are still many species of fungi whose plant growth-promoting properties have been understudied or not studied at all. We examined the growth-promoting effect in spring barley (*Hordeum vulgare*) and Italian ryegrass (*Lolium multiflorum*) induced by three endophytic fungi previously obtained from the roots of *Festuca*/*Lolium* grasses. Surface-sterilized seeds were inoculated with a spore suspension of *Cadophora fastigiata* (isolate BSG003)*, Paraphoma fimeti* (BSG010), *Plectosphaerella cucumerina* (BSG006)*,* and their spore mixture. Before harvesting, the inoculated plants were grown in a greenhouse, with the barley being in multi-cavity trays for 30 days and ryegrass being placed in an original cylindric element system for 63 days. All three newly tested fungi had a positive effect on the growth of the barley and ryegrass plants, with the most pronounced impact observed in their root size. The fungal inoculations increased the dry shoot biomass between 11% and 26% in Italian ryegrass, but no such impact was observed in barley. The highest root increment was observed in barley. Herein, *P. cucumerina* and *C. fastigiata* inoculations were superior to other treatments, showing an increase in root dry weight of 50% compared to 20%, respectively. All fungal inoculations significantly promoted root growth in Italian ryegrass, resulting in a 20–30% increase in dry weight compared to non-inoculated plants. Moreover, a strong stimulatory effect of the fungi-emitted VOCs on the root development was observed in plate-in-plate arrays. In the presence of *C. fastigiata* and *P. cucumerina* cultures, the number of roots and root hairs in barley seedlings doubled compared to control plants. Thus, in our study, we demonstrated the potential of the grass root-derived endophytes *C. fastigiata*, *P. fimeti*, and *P. cucumerina* as growth promoters for spring barley and Italian ryegrass. These studies can be extended to other major crops and grasses by evaluating different fungal isolates.

## 1. Introduction

Mutualistic relationships between plants and endophytic fungi have attracted significant interest in recent decades [1,2,3]. Studies of agricultural crops, such as wheat, barley, soybean, corn, rice, cotton, and others, reveal the species diversity of endophytic fungi and their importance to host plants [4,5]. Many studies highlight the positive aspects of endophytic fungi, showing increased resistance to abiotic and biotic stress and acting against insect pests and plant pathogens [6,7]. Along with the benefits listed above, more and more endophytic fungi are confirmed to act as plant growth stimulants with the possibility of being used in agricultural practice, reducing the need for chemical fertilizers, as well reviewed in several recent papers [8,9,10,11,12]. Although the path between the laboratory and practical use is long and complex, this interface is increasingly being fulfilled. For example, in spring barley field trials, a commercial inoculant Bioagro^®^ Grass (Glenside Co., Stirling, Scotland, UK) based on *Rhizophagus* sp. arbuscular mycorrhizal fungi has been shown to improve P uptake, plant growth, and grain yield [13]. In addition, the study by Nauanova et al. [14] demonstrated that a *Trichoderma* sp. -based inoculant, originating from the soils of North Kazakhstan, had a positive effect on increasing the barley grain yield by 50% and exceeding the effect of other inoculants based on bacterial strains.

In the course of plant–fungal interaction studies, the growth-promoting effect has been widely determined for the fungi of two particular groups. One group is arbuscular mycorrhizal fungi (AMF), members of Glomeromycota, which are highly abundant plant symbionts found in many plants. These fungi promote different aspects of plant life, showing improved nutrition and better growth, stress tolerance, and disease resistance [15]. Another group is entomopathogenic fungi (*Metarhizium*, *Beauveria*, *Purpureocillium*), which have been found to exhibit growth-stimulatory properties in the experiments when they were investigated as pest control agents. Meanwhile, some *Trichoderma* (Hypocreales, Sordariomycetes) species that fall out of these two groups are also well known for their growth-promoting effects [16]. Hence, many studies show that *Glomus* [14], *Trichoderma* [17,18], *Metarhizium* [19,20,21], and *Beauveria* [22] fungi have beneficial impacts on plant shoot and root growth in diverse plant species.

The benefits of fungal colonization have been shown in grain crops and forage grasses. *Lolium multiflorum* seed inoculation with *Trichoderma harzianum* demonstrated a beneficial impact on seed germination and seedling growth along with antifungal suppression against seed-borne fungi [23]. Inoculation of wheat seeds with endophytic fungal entomopathogens *Beauveria* and *Metarhizium brunneum* improved plant growth and reduced crown and root rot caused by *Fusarium culmorum* [21]. Also, in wheat, an inoculant of the mix of eight arbuscular mycorrhizal fungi species added to soil in pots positively influenced root biomass and root density and increased the uptake of P, Fe, and Zn [24].

Recently, the list of growth-promoting fungi species has been extended in view of the new results regarding searching for fungal biostimulants. *Penicillium*, *Aspergillus*, *Rhizoctonia*, *Fusarium*, and *Chaetomium*, among others, have been specified as growth promoters in different crops, such as tomato, cucumber, maize, and rice [10]. However, there are still many endophytic fungi with little or no research on plant growth-promoting properties. In addition, little research has been conducted on forage grasses and spring barley that are grown worldwide and provide nutrition for livestock and humans.

In our study, we have chosen three endophytic fungi that were not previously studied for their impact on plant growth. These are *Cadophora fastigiata* (isolate BSG003), *Paraphoma fimeti* (BSG010), and *Plectosphaerella cucumerina* (BSG006); they were obtained from the roots of *Festuca*/*Lolium* grasses by our research group ([25] for fungal strain identification procedures). These three fungi were selected from our laboratory collection because they are not pathogenic to spring barley and Italian ryegrass (no data in the literature) and due to the spore production in the culture being sufficient (our laboratory test). All three fungi, although taxonomically coming from different orders, are widely found in soil and as plant endophytes. They inhabit grassland and woodland ecosites, acting as saprotrophs with an important role in decomposing organic matter, and they are also found endophytically. *C. fastigiata* is a common root endophyte across very different plant species; however, it has been specified to be more linked to tree/wood [26,27] than to grassland habitats [1,28,29]. *P*. *cucumerina* is well studied in its association as a pathogen of many horticultural plants [30,31]. *P. fimeti* is the least studied of all, but it has been repeatedly found in soil and endophytically worldwide [32,33,34].

This work aimed to test the growth-promoting properties of *C. fastigiata*, *P. fimeti*, and *P. cucumerina* in spring barley (*Hordeum vulgare* L.) and Italian ryegrass (*Lolium multiflorum* Lam.). We were interested in investigating the inoculation of *C. fastigiata* in Italian ryegrass since *Cadophora* sp. was earlier shown to be a growth-promoter in perennial ryegrass (*Lolium perenne*) by Berthelot et al. [35,36]. The other two fungi were chosen because we obtained them as endophytic colonizers of grass roots, which is potentially beneficial for plant development, but they do not have sufficient evaluation records yet for growth-promoting abilities. Firstly, we tested the growth-promoting effect in spring barley, a fast-growing annual grain crop, in a multi-cavity trays experiment for 30 days. Next, we extended the growth-promoting assessment to Italian ryegrass, a biannual forage grass, in an originally designed structure of cylinder elements for 63 growth days. The inoculated plants of two species were assessed for their shoot and root biomass. In addition, we carried out an investigation of spring barley roots exposed to fungal culture-emitted VOCs in vitro. From our results, for the first time, we report on the growth-promoting properties of *C. fastigiata*, *P. fimeti*, and *P. cucumerina* revealed in two agricultural crops, namely spring barley and Italian ryegrass.

## 2. Materials and Methods

### 2.1. Plant Material and Seed Sterilization

Spring barley var. Gunda (Lithuanian Centre for Agricultural and Forestry Sciences, Institute of Agriculture, Akademija, Kėdainiai distr., LT) and Italian ryegrass var. Druva (State Priekuli Plant Breeding Institute, Priekuli, LV) seeds were used for plant growth assessments. A laboratory protocol was used for sterilization. In order to break the seed coat and increase the efficiency of inoculation, the seeds were treated with 50% sulfuric acid (diluted with water) (Thermo Fisher Scientific Baltics, Cat. No. 124640011, Vilnius, Lithuania) for 10 min, rinsed twice with sterile water, and surface-sterilized as follows: 70% ethanol for 2 min, sterile water wash, ½ dilution of 5% sodium hypochlorite (Thermo Fisher Scientific Baltics, Cat. No. P005-03, Vilnius, Lithuania) for 10 min, three times sterile water wash, last wash for 10 min.

### 2.2. Fungal Isolates

We used the following fungal isolates from our laboratory collection: *C. fastigiata* isolate BSG003 was obtained from *Festuca gigantea*, *P. fimeti* BSG010 and *P. cucumerina* BSG006 from *Lolium perenne* × *F. gigantea* hybrids (laboratory plant collection). The fungal strain isolation procedures were as described in Pašakinskienė et al. [25]. The taxonomic assignment was based on the colony morphology and the cytomorphological characteristics of the species and confirmed by the alignment of the PCR-produced ITS, *RPB2*, *SSU*, and *TEF1-a* sequences with the reference fungal DNA data in NCBI [25].

### 2.3. Seed Inoculation

For seed inoculation, *C. fastigiata*, *P. fimeti* and *P. cucumerina* spore suspensions were prepared from 14-day-old cultures on the PDA medium. Fifteen Petri plates were prepared for each fungus strain. Four ml of sterile water were dispensed on each Petri plate; spores were spread and collected in sterile tubes by gently brushing them with the tip of an automatic pipette (Thermo Fisher Scientific Baltics, Cat. No. P005-03, Vilnius, Lithuania). Spore density was calculated by hemocytometer (Precicolor HBG, Giessen, Germany) according to the recommendations of [37] and stained by Trypan Blue. Quantification was conducted in triplicate (*n* = 3) for statistical accuracy. Spore concentrations were calculated in spores per milliliter (spores/mL) to ensure clarity and reproducibility. The spore inocula were adjusted to ~3.5–4 × 10^5^ per ml density. The mixture of the three fungal strains was prepared to a ratio of 1:1:1. About 50 mL of a spore suspension was made for each inoculation treatment. A laboratory protocol was applied for inoculation as follows: after sterilization, the seeds (N = 150 for barley and N = 100 for ryegrass) were mixed with endophytic fungal spore suspension and the tubes were placed in a rotor shaker (Eppendorf SE, Hamburg, Germany) at 140 rpm at 28 °C for 30 min. After the inoculation treatment, the seeds were briefly rinsed with sterile water. Sterilized seeds (Section 2.1) without spore suspension treatment were used as a control.

### 2.4. Spring Barley Growth in Multi-Cavity Trays

The inoculated and control (non-inoculated) seeds were germinated at room temperature (22–24 °C) in Petri dishes on sterile paper moistened with sterile water. After 7 days, the germinated barley seeds were planted in multi-cavity trays (28 positions of 6.5 × 6.5 × 6.2 cm) filled with an autoclaved (121 °C, 30 min) mixture of peat and compost soil at a ratio of 1:4. Peat composition: raised bog peat, dolomite, sphagnum; humidity up to 70%, acidity 5.5–6.5, nutrients, mg/L: N 100–400, P 50–200, K 100–500. Compost soil composition: humidity—not more than 60%, acidity 5.5–6.5, organic matter—93%; total nitrogen (N)—0.2, total phosphorus (P2O5)—0.007%, total potassium (K)—0.05%.

For each of the four inoculation treatments and control, N = 54 barley seedlings were planted and grown in a greenhouse in controlled light with a 16/8 h photoperiod (200–220 μmol/m^2^/s) and a temperature of 24–26 °C/16–18 °C (day/night). After 30 days of growth, the aerial parts and roots of the plants were harvested. The shoot green biomass was weighed immediately after harvesting. For root dry biomass assessment, the roots were washed of the soil and dried at ~35 °C for 48 h.

### 2.5. Italian Ryegrass Growth in a Cylinder Elements System

For the inoculation of *L. multiflorum* ‘Druva’ seeds, the spore suspensions of *C. fastigiata*, *P. fimeti*, and *P. cucumerina* 14-day isolates were used. The inoculation procedure was the same as described for barley (Section 2.3). Germinated seeds were pre-grown for 2 weeks in multi-cavity trays in the greenhouse. Later, well-established seedlings, N = 30 for each treatment and control, were transplanted into h50 × Ø11 cm plastic cylinders (two plants per cylinder) filled with a growth substrate, which was a 1:4:1 mixture of peat, compost soil, and perlite autoclaved at 121 °C for 30 min. In this original construction of cylindric elements, hand-made geotextile fabric bags fitting the cylinder volume were used. Fifteen cylindric elements for each inoculation treatment and control were arranged in 35 × 65 × h30 cm plastic containers, numbering 5 in total. Holes were punched at the bottom of the textile bags before filling them up with the growth substrate, and plastic Honeycomb panels were placed in the bottom of the container to have the water properly drained. The plants were watered twice a week with 115 mL for 2 weeks, twice a week with 330 mL of water, and then, for the last three weeks, with 370 mL of water twice a week. The plants were grown in September–October 2023 in a polypropylene-covered greenhouse under conditions of natural light and temperatures of 24–28 °C/12–16 °C (day/night).

After 63 days of post-inoculation growth, the plant shoots and roots were harvested and evaluated. The root length and shoot height were measured and the number of shoots was counted. The shoot green biomass was weighed immediately after harvesting. For shoot and root (washed of the soil) dry biomass assessment, the samples were dried at ~35 °C for 48 h.

### 2.6. Spring Barley Seedlings Exposed to Fungal Cultures VOCs in Plate-In Plate Arrays

The cultures of endophytic *C. fastigiata* and *P. cucumerina* fungi were grown in 35 mm diameter Petri plates on the PDA medium supplemented with ampicillin (final concentration—100 µg/mL) and streptomycin sulfate (final concentration—100 µg/mL) at 27 °C in the dark for two days. These small plates with the fungus cultures were placed in large, 120 × 120 mm, square Petri dishes filled with a Murashige and Skoog medium prepared at ½ of standard concentration (without additional sucrose and vitamins). Then, sterilized barley seeds (as described in Section 2.1) were placed for growth in square Petri dishes at 22–24 °C, 16/8 h photoperiod. In these plate-in-plate arrays, the barley seedlings were exposed to fungal culture VOCs. After 5, 6, and 7 days of growth, the number of main and lateral roots was counted. The number of root hairs was evaluated under the Euromex NexiusZoom EVO stereomicroscope (Cat. No. NZ.1902-B, Euromex Microscopen, Duiven, The Netherlands) using the ImageFocusAlpha (version 4) program. For each treatment and control (arrays without the fungal culture), areas of 0.50 mm^2^ in 10 roots were assessed.

### 2.7. Statistical Analysis

Statistical analysis was performed using STATISTICA^®^ 7.0. A statistical assessment was carried out by a Student’s *t*-test. The fungal effects on the plant growth parameters were analyzed with a one-way ANOVA. The statistical significance of the differences between the means was assessed by a post hoc Tukey’s test. Differences were considered to be significant at *p* ≤ 0.05. Charts were drawn using MS Excel software 2016.

## 3. Results

### 3.1. Inoculated Spring Barley Growth in a Multi-Cavity Tray Experiment

Barley, like the *Lolium* species, belongs to the Poaceae family. An advantage of barley in the experiments with fungal inoculation is its much faster growth. Thirty days of growth is sufficient to assess changes, whereas experiments with grasses take at least 2 months for fast-growing annual species and 6 months for perennials. This aspect is important when new fungi species are tested for a possible growth-promoting effect.

Spring barley ‘Gunda’ seeds were inoculated with the fungal spore suspensions of the isolates obtained from the roots of *Festuca*/*Lolium*, namely *C. fastigiata* BSG003, *P. fimeti* BSG010, *P. cucumerina* BSG006, and their mixture. Fungal colonization was found to be successful; >90% of roots examined (N = 50 per treatment, Trypan Blue staining as in Section 2.3) had hyphal fungal structures at day 4 post-inoculation in all inoculation variants (~30% in control).

The inoculations increased the seed germination rate in spring barley. The germination rate in the inoculation treatments ranged from 73.3% to 93.0%, whereas, in the control group (sterilization without inoculation), it was 63.0%.

**Figure 1:**
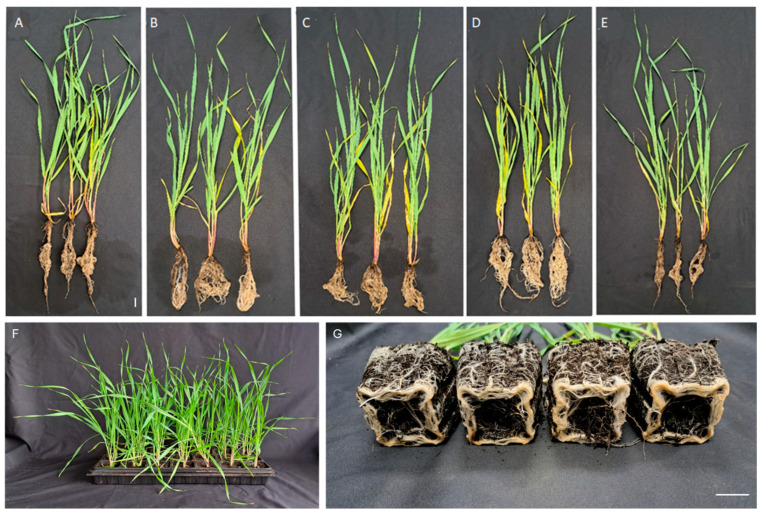
Spring barley plants affected by fungal inoculations after 30 days of growth in multi-cavity trays. (**A**) Control plants, no fungal inoculation; (**B**–**E**) inoculations as follows: *Cadophora fastigiata* BSG003 (**B**), *Paraphoma fimeti* BSG010 (**C**); *Plectosphaerella cucumerina* BSG006 (**D**); the three fungi mix (**E**); (**F**,**G**) representative views of the plants from *P. cucumerina* treatment in a multi-cavity tray (**G**), and a root display from the bottom (**F**). Scale bar 3 cm.

**Figure 2:**
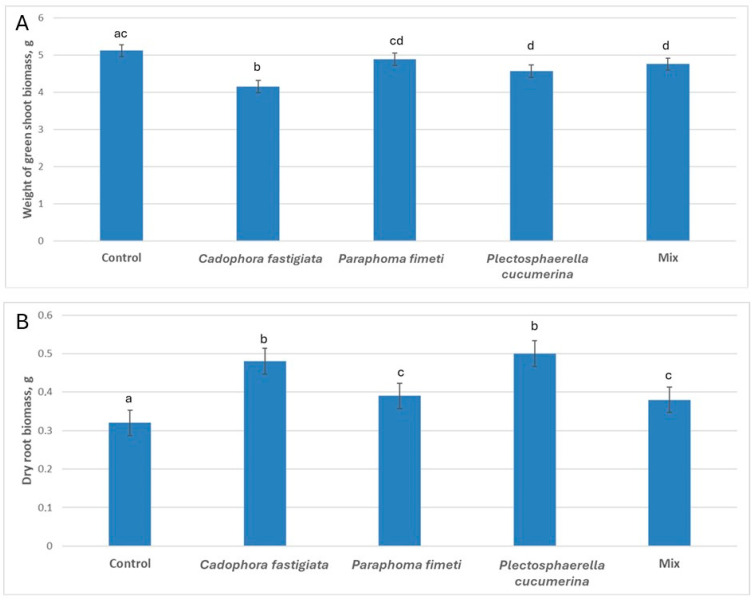
Spring barley growth parameters affected by fungal inoculations after 30 days of growth in multi-cavity trays. (**A**) green shoot biomass; (**B**) dry root biomass. Different letters (a, b, c and d) above the bars indicate significant differences between the treatments (*p* ≤ 0.05) based on Tukey’s HSD post hoc test.

The assessment of the shoot green biomass and root dry biomass of the barley plants was carried out after 30 days of post-inoculation growth in multi-cavity trays in a sterile soil substrate in the greenhouse (Figure 1F). Fungal spore inoculations had affected the plant growth. The shoot biomass of the inoculated plants was slightly reduced as compared to untreated plants, excluding *P. fimeti* treatment, where no significant difference from the untreated plants was found (Figure 2A). In contrast, the root volume measured by the dry weight significantly increased in all inoculations, and *P. cucumerina* and *C. fastigiata* were superior (56% and 50% increment, respectively) to other treatments, namely *P. fimeti* and the inoculant mix (22% and 19% increment, respectively) (Figure 1A–E and Figure 2B). In the inoculation treatments with *C. fastigiata* and *P. cucumerina*, where root growth enhancement was superior, the shoot biomass had a slight tendency to decrease compared to the untreated plants (Figure 2A,B). We assume that, due to a small volume of soil in the multi-cavity trays, the balance of the plant development has been modified in favor of root growth (Figure 1G).

### 3.2. Inoculated Italian Ryegrass Growth in a Cylinder Elements System

**Figure 3:**
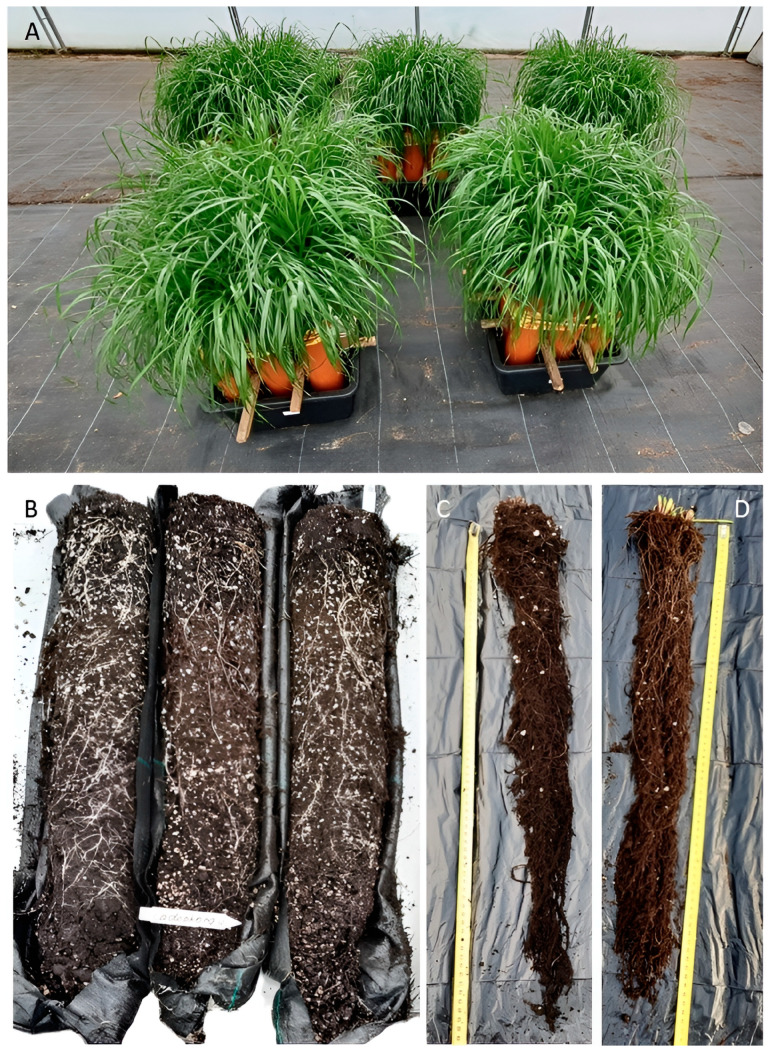
A view of the experimental panel of Italian ryegrass plants affected by fungal inoculations after 63 days of growth in the cylinder elements system. (**A**) A representative view of five blocks of plants from *Cadophora fastigiata* BSG003, *Paraphoma fimeti* BSG010, *Plectosphaerella cucumerina* BSG006, the three fungi mix inoculations and the control; (**B**–**D**) representative views of the plants from *C. fastigiata* treatment: roots in the soil at the moment of the opening of geotextile bags (**B**), control plant roots measured (**C**) next to the plant roots from *C. fastigiata* inoculation (**D**).

Following the positive growth-promoting results of the spring barley treatment, the second experiment was designed to test the inoculation effect on the plant growth of *L. mutiflorum*, a fast-growing biannual grass. An original system of cylinder elements (Ø11 cm × h50 cm) was developed in which plants were grown for 63 days in a sterile soil substrate in the greenhouse, followed by shoot and root harvesting (Figure 3A–D).

**Figure 4:**
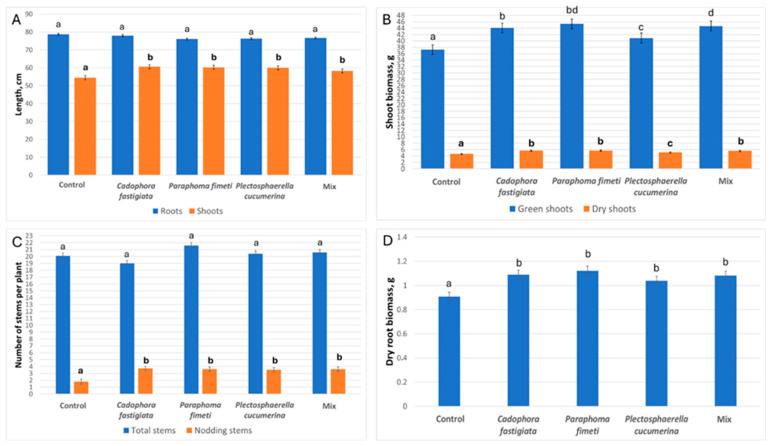
Italian ryegrass growth parameters affected by fungal inoculations after 63 days of growth in the cylinder elements system. (**A**) Shoot and root height; (**B**) shoot green and dry biomass; (**C**) shoot number; (**D**) dry root biomass. Different letters (a, b, c and d) above the bars indicate significant differences between the treatments (*p* ≤ 0.05) based on Tukey’s HSD post hoc test. In (**A**), regular font for root length, bold—for shoot height; in (**B**), regular—for green shoot weight, bold—for dry shoot weight; in (**C**), regular—for total stem no., bold—for nodding stem no.

The inoculations increased the seed germination rate in Italian ryegrass. The seed germination rate ranged from 78% to 82% in the inoculated variants, whereas, in the control group (sterilization without inoculation), it was 68%. Inoculation with endophytic fungi resulted in a ~10% increase in the shoot height of Italian ryegrass across all variants, while root length showed no significant difference compared to the control (Figure 4A). There was no significant difference between the total number of shoots in different inoculation groups and the control group (Figure 4C). However, a significant increment was observed in all inoculation treatments for the number of nodding shoots per plant, i.e., 3.5 nodding shoots out of ~20 in total compared to 1.8 in control. This indicates that the inoculated plants had a faster development and were approaching the transition from vegetative growth to flowering.

Inoculation with all fungi had a significant stimulating impact on the biomass of the shoots and roots of the Italian ryegrass. The biomass of fresh and dry shoots increased by between 20 and 26% in the *C. fastigiata*, *P. fimeti*, and spore mixture treatments and by 11% in the *P. cucumerina* variant (Figure 4B). Thus, in *P. cucumerina* inoculation, the stimulating effect for shoot biomass was found to be less pronounced than in other inoculations. All fungal inoculation treatments significantly promoted root growth, resulting in a 20–30% increase in dry weight compared to non-inoculated plants (Figure 4D). Although the treatment with the mixture of the three fungi showed a growth-promoting effect, it was not superior to the best single-isolate inoculations (Figure 4A–D).

### 3.3. Root Growth of Spring Barley Exposed to Fungal Culture VOCs in Plate-in-Plate Assays

Spring barley seedlings were grown in large square plates of 120 × 120 mm on a Murashige–Skoog nutrient medium next to endophytic fungal isolates on a PDA medium in small Ø35 mm plates. In a closed environment created in such a plate-in-plate system, the barley seedlings were exposed to volatile organic compounds (VOCs) released by the fungi. The isolates of two species of endophytic fungi were tested, namely *C. fastigiata* BSG003 and *P. cucumerina* BSG010.

**Figure 5:**
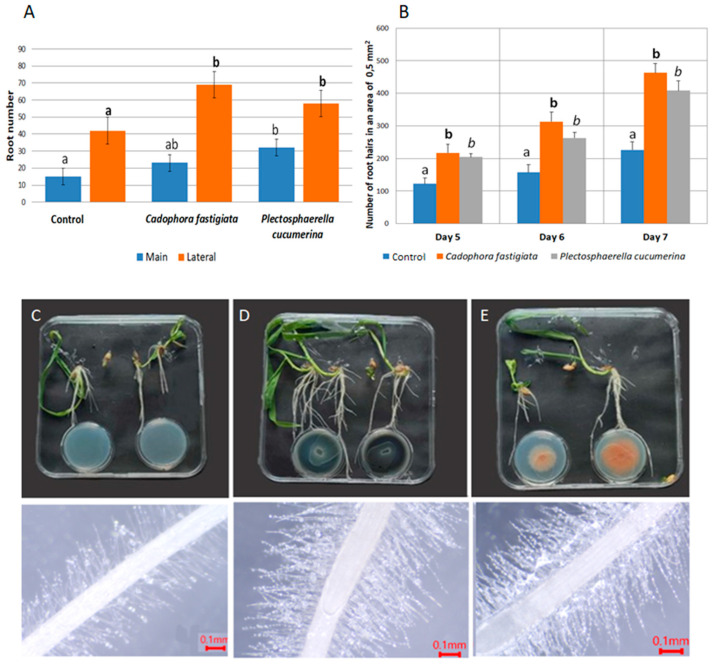
The effect on of barley root development exposed to VOCs of endophytic fungi *Cadophora fastigiata* BSG003 and *Plectosphaerella cucumerina* BSG006. (**A**) The effect on the number of main and lateral roots after 7 days of growth; (**B**) the effect on the number of root hairs on the 5th, 6th and 7th days in a 0.5 mm^2^ area. (**C**–**E**) Spring barley plants grown in plate-in plate assays (top images) and microscopical images of the roots (bottom images) under the exposure of fungal cultures: (**C**) control, no fungal culture in small plates, (**D**) barley with *C. fastigiata* BSG003, (**E**) barley with *P. cucumerina* BSG010. Different letters (a, b, c and d) above the bars indicate significant differences between the treatments (*p* ≤ 0.05) based on Tukey’s HSD post hoc test. In (**A**), regular font for main root no., bold—for lateral root no.; in (**B**), regular—for control, bold—for *C. fastigiata*, italicized—for *P. cucumerina*.

The assessment of main and lateral roots revealed that exposure to VOCs released by *C. fastigiata* and *P. cucumerina* had a strong stimulating impact on root development (Figure 5). After 7 days of growth, the root number significantly increased in both treatments. The highest number of main roots in barley was observed in assays with *P. cucumerina*, and the highest number of lateral roots was observed in the *C. fastigiata* treatment, with 110% and 70% increments from the control, respectively (Figure 5A).

In addition, root hair numbers were evaluated microscopically on the 5th, 6th, and 7th days of growth. The ImageFocusAplha program was used for calculations in the 0.5 mm^2^ root segment area. Root hair numbers significantly increased in both *C. fastigiata* and *P. cucumerina* fungal VOC treatments (Figure 5B). By the 7th day, the root hair number in terms of both *C. fastigiata* and *P*. *cucumerina* exposure had doubled compared to the VOC-untreated plants. The stimulating effect on hair number was already evident by the 5th day of growth (early germination). This suggests that fungal VOC stimulation for barley roots is immediate, as we have determined in plate-in-plate assays.

In our study, for the first time, the isolates of three endophytic fungi *C. fastigiata* (BSG003), *P. fimeti* (BSG010), and *P. cucumerina* (BSG006), originating from the roots of Festuca/Lolium grasses ([25] for *C. fastigiata*, and unpublished data for others), were evaluated for their growth-promoting effects. For barley, plants were harvested after a 30-day post-inoculation growth in multi-cavity trays, and, for Italian ryegrass, after a 63-day growth in cylindrical tubes. In the soil growth experiments, the fungi positively affected various growth parameters: the plant height, green/dry shoot, dry root biomass in Italian ryegrass, and dry root biomass in spring barley. When comparing different fungi and their spore mixtures, all fungal inoculations effectively promoted root growth in Italian ryegrass, showing a 20–30% increase in dry weight, with no statistically significant differences being observed between the fungal treatments, whereas, in spring barley, the root growth stimulation by *C. fastigiata* and *P. cucumerina* showed a greater impact, resulting in a 50% and 56% increase in dry root weight, respectively. In the plate-in-plate assays, where the plants were exposed to fungi-culture-emitted VOCs, a strong stimulating effect of *C. fastigiata* and *P. cucumerina* on spring barley root development was revealed. This was shown by doubling the number of roots and the proliferation of root hairs. Overall, the effect of promoting root growth was more pronounced than that of shoot growth, especially in the barley multi-cavity tray experiment. From these results, we propose spring barley, a fast-growing annual crop, as a host plant for an indicative-testing method to investigate the growth-promoting effects of fungi inoculations. The special cylinder elements system that we have developed for Italian ryegrass post-inoculation growth can be used in plant–fungi interaction studies for many grasses and some woody plants.

## 4. Discussion

The examples of fungal growth-promoting effect evaluation in spring barley and Italian ryegrass are so far limited as compared to other well-studied plant species, such as *Arabidopsis thaliana*, *Zea mays*, wheat, tomato, and rice, among others [9,13,24,38]. Previously, in ryegrass, a growth-stimulating effect was demonstrated by inoculation with *Rhodotorula* spp. and their mixture with endophytic bacteria isolated from poplar and willow [39], *Cadophora* sp. obtained from poplar at metal-polluted sites [35,36], *Trichoderma harzianum* [23], and *Xerocomus badius* and *Serendipita indica* [40]. In barley, *Piriformospora indica* [41], *Trichoderma* sp. [42,43], arbuscular mycorrhizal fungi [44], salt tolerant fungi *Periconia macrospinosa*, *Neocamarosporium goegapense*, *N. chichastianum* [45], and *Epichloë bromicola* [46] have been shown to benefit plant growth.

For the growth-promotion evaluation, we chose species of endophytic Ascomycota previously isolated from the roots of *Festuca*/*Lolium* plants (laboratory isolates’ collection). The isolates of three endophytic fungi, *C. fastigiata* (isolate BSG003), *P. cucumerina* (BSG006), and *P. fimeti* (BSG010) ([25] for *C. fastigiata*, unpublished data for others) were evaluated for their growth-promoting effect in spring barley and Italian ryegrass. Until now, there were no data on *P. cucumerina* and *P. fimeti* inoculations for the growth-promotion assessment, whereas some *Cadophora* inoculations have been carried out without specifying fungal species [35,36,47]. We found that inoculation with all three fungi had increased Italian ryegrass plant height and green/dry shoot biomass, and the shoot biomass increment depended on the fungal species. Moreover, all three fungi significantly improved the root growth of Italian ryegrass and spring barley. All fungal inoculations demonstrated similar effectiveness in enhancing root growth in Italian ryegrass, whereas, in spring barley, the root growth stimulation by *C. fastigiata* and *P. cucumerina* showed a greater impact. Furthermore, when the spring barley seedlings were exposed to VOCs released by the fungal cultures, we observed a strong stimulatory effect of *C. fastigiata* and *P. cucumerina* on the root development, as indicated by a double increase in the root number and proliferation of root hair.

The greater beneficial impact of fungal inoculation on root growth compared to shoots was highlighted in many studies regarding grasses. For example, under drought exposure, when applying *Rhodotorula* spp. inoculations and their mixture with endophytic bacteria, the root growth increment surpassed the shoot biomass increase in *L. perenne* (60% and 48%, respectively) [39]. Also, in this study, perennial ryegrass was found to be the best choice as a host plant for inoculations compared to the other tested grasses, Kentucky bluegrass (*Poa pratensis*), bent grass (*Agrostis*), and hair grass (*Deschampsia*), which did not respond well to fungal inoculations [39]. Moreover, the study by Berthelot et al. has shown that *Chadophora* sp. inoculation doubled the root dry weight of *L. perenne*, whereas the shoot weight increase was not statistically significant [35]. A doubled effect for root number was also observed in Italian ryegrass inoculated with *Trichoderma harzianum* along with the suppression of soil-borne fungi, *Alternaria alternate* and *A. ventricosa* [23].

A greater beneficial impact on root growth stimulation was also determined in the experiment with barley under the inoculations of *Trichoderma* sp., >100% and 60% increments, respectively, accompanied by a 40% increase in grain yield [42]. In the complex study with three salt-tolerant fungi inoculations, the increments of barley shoot and root biomass were found to be similar, reaching the maximum effects of a 50–60% increase under exposures to different drought and salinity levels [45].

Many samples of the root endophyte *Cadophora fastigiata Lagerb. & Melin* [=*Phialophora fastigiata* (Lagerb. & Melin) Conant] (Helotiales), which is a type of *Cadophora* species, come from different plant species [1,25]. However, its distribution is more commonly observed in tree and/or woody habitats. Like many other *Cadophora* [26,27,48], *C*. *fastigiata* has often been found growing on wood pulp and tree roots [28,49]. There are several records showing that *Cadophora* spp. can act as a biostimulant and biocontrol agent. For example, Yakti et al. [47] identified various biostimulant effects of *Cadophora* sp., including increasing tomato root and shoot biomass. In addition, the isolates of *Cadophora* sp. were shown to act against the wilting caused by *Fusarium* sp. in *Cucumis melo* plants [50]. For perennial ryegrass (*Lolium perenne*), Berthelot et al. [35,36] revealed the stimulating plant-growth effect of *Cadophora* sp. inoculation in the experiment with seven isolates of different Helotiales (*Cadophora*, *Leptodontidium*, *Phialophora* and *Phialocephala*) obtained from the roots of poplar trees grown in metal-polluted sites. In their study, the inoculation with *Cadophora* sp. Fe06 isolate remarkably enhanced *L. perenne* root growth compared to other fungal strain treatments. This agrees with our results demonstrating that *C. fastigiata* inoculation significantly stimulates the root growth of spring barley (50% increment) and Italian ryegrass (25% increment). Furthermore, Berthelot et al. [35,36] observed that, for plant growth-promotion effects, *L. perenne* plants show a stronger response to fungal inoculations compared to birch and eucalyptus, and these treatments were found to be particularly beneficial for root growth. This suggests that *Lolium* grasses are suitable as host plant species for testing the growth-promoting efficacy of fungal isolates. There are no data on *Cadophora* sp. inoculation assessments in barley. However, an increase in barley root growth was achieved due to colonization by the well-studied fungus *Trichderma* sp. This resulted in a higher plant shoot biomass and grain yield due to better nutrient and water uptake [42,43].

*Plectosphaerella cucumerina* (Lindf.) W.Gams (=*Fusarium tabacinum* (J.F.H.Beyma) W.Gams) (Glomerellales) is a soil fungus, a decomposer of plant organic matter, and a common root pathogen with a wide range of host plants [31,51]. *P. cucumerina* is a pathogen of many Cucurbitaceae, causing plant collapse and root rot infections [30]. This fungus also causes damage to tomatoes, peppers, asparagus, cabbage, and potatoes [51]. However, no data indicate that *P. cucumerina* could infect and cause diseases in Poaceae plants. Several studies show that *P. cucumerina* can act as a biological control agent. For example, *P. cucumerina* has demonstrated nematophagous activity against potato cyst nematodes and was found to be efficient in reducing the pest field populations in UK trials [52,53]. Interestingly, *P. cucumerina* has also been identified as a potential bioherbicide for controlling *Cirsium arvense* weed in Canada and New Zealand [54]. However, no information is available on *P. cucumerina* acting as a plant growth promoter. In our study, a significant *P. cucumerina* root growth-promoting impact was revealed in spring barley (56% increment) and Italian ryegrass (20% increment). In addition to the root growth stimulation, a 10% increment was revealed for Italian ryegrass shoot biomass after *P. cucumerina* inoculation. In summary, our study, for the first time, revealed the plant growth-promoting effects of *P. cucumerina* inoculations by the assessment of two agricultural crops, namely spring barley and Italian ryegrass.

*Paraphoma fimeti* (Brunaud) Gruyter, Aveskamp & Verkley (=*Phoma fimeti* Brunaud) (Pleosporales) is a common member of the genus of soil fungi with a diverse ecological lifestyle. *P. fimeti* is often sampled from the dead tissue of herbaceous and woody plants. The taxon *Paraphoma* was previously included in the genus *Phoma* as a section [32,55], but later it was confirmed as a separate genus according to typical characteristics of glabrous pycnidia and dictyochlamidospores [56,57]. Several pathogenic *Paraphoma* species have been reported to cause crown rot and root infections in Compositae plants [58]. However, there is no evidence of *P. fimeti* colonizing grasses and cereals, and the plant growth-promoting effect of *P. fimeti* has not so far been documented according to the literature. In our study, *P. fimeti* was efficient in promoting Italian ryegrass shoot and root growth, with 26% and 30% increments being seen in dry weight, respectively. Among the fungi tested, *P. fimeti* and *P. cucumerina* performed the best in terms of enhancing the shoot and root growth of Italian ryegrass. However, in spring barley, the root growth stimulatory effect of *P. fimeti* isolate was less efficient compared to other fungal treatments.

The data on the beneficial effects of fungal VOCs are currently rapidly accumulating, demonstrating the stimulatory properties of VOCs released by endophytic fungi in the regulation of symbiotic associations [59], promotion of plant defense against phytopathogens [60], stimulation of plant growth [38,61] and alleviation of abiotic stress [62]. In our study, we investigated the VOC effects of *C. fastigiata* and *P. cucumerina* cultures for the first time and observed a significant stimulation of spring barley root development in vitro. It is known that fungal VOCs can reprogram the root architecture of plants using different strategies and stimulate their growth, thereby benefiting from an increased colonization surface [63,64]. On the other hand, the increased surface area of roots, especially root hairs, gives the host plant the advantage of better access to necessary nutrients. Overall, the VOC-induced growth-promoting effects on plant roots and root hairs appear to be a widespread phenomenon caused by a wide range of fungal isolates [65,66,67,68,69]. Additionally, *Trichoderma* spp. VOCs have shown strong antifungal properties, significantly inhibiting pathogenic fungi such as *Alternaria panax*, *Botrytis cinerea*, and *Sclerotinia nivalis* while also improving plant growth [16,70]. These studies highlight the promising role of VOCs as both biocontrol agents and enhancers of plant health in agriculture.

## 5. Conclusions

Given the prevalence of endophytic fungi, there is a great need to evaluate their ability as plant growth promoters. In our study, for the first time, the isolates of three endophytic fungi, *C. fastigiata* BSG003, *P. fimeti* BSG010 and *P. cucumerina* BSG006, previously obtained from the roots of *Festuca*/*Lolium* plants, were shown to have growth-promoting effects in spring barley and Italian ryegrass. The growth enhancement impact on the roots of both plants was more pronounced compared to the shoots. A stimulatory effect on root growth was also confirmed in vitro, demonstrating the impact of VOCs released by fungal cultures of *C. fastigiata* BSG003 and *P. cucumerina* BSG006. Both spring barley and Italian ryegrass proved to be well responding host plants in the evaluation of the growth-promoting effects of fungal culture inoculations. In the future, such evaluations will allow us to speed up the fungal isolate screening and help extend research schemes to other major cereals and grasses. In summary, our findings contribute to the knowledge regarding endophytic fungi as biostimulants of plant growth. However, further research is needed to link these data to the schemes of biofertilization in agricultural crops.

## Data Availability

All data are contained within the article.
